# Minimal Linear Codes Constructed from Sunflowers

**DOI:** 10.3390/e25121669

**Published:** 2023-12-18

**Authors:** Xia Wu, Wei Lu

**Affiliations:** School of Mathematics, Southeast University, Nanjing 210096, China; wuxia80@seu.edu.cn

**Keywords:** linear code, minimal code, sunflower, 94B05, 94A62

## Abstract

Sunflower in coding theory is a class of important subspace codes and can be used to construct linear codes. In this paper, we study the minimality of linear codes over $F_{q}$ constructed from sunflowers of size *s* in all cases. For any sunflower, the corresponding linear code is minimal if s≥q+1, and not minimal if 2≤s≤3≤q. In the case where 3<s≤q, for some sunflowers, the corresponding linear codes are minimal, whereas for some other sunflowers, the corresponding linear codes are not minimal.

## 1. **Introduction**

Let $F_{q}$ be the finite field with *q* elements and $F_{q}^{n}$ the vector space with dimension *n* over $F_{q}$. For a vector $v=\left(v_{1},\ldots ,v_{n}\right)\in F_{q}^{n}$, let Suppt(v)$:=\{1\leq i\leq n:v_{i}\neq 0\}$ be the support of v. The *Hamming weight* of v is wt(v):=#Suppt(v). For any two vectors $u,v\in F_{q}^{n}$, if Suppt(u)⊆Suppt(v), we say that v covers u (or u is covered by v) and write u⪯v. Clearly, av⪯v for all $a\in F_{q}$.

An ${\left[n,m\right]}_{q}$ linear code C over $F_{q}$ is an *m*-dimensional subspace of $F_{q}^{n}$. A codeword c in a linear code C is called *minimal* if c covers only the codewords ac for all $a\in F_{q}$, but no other codewords in C. If every codeword in C is minimal, then C is said to be a *minimal linear code*. Minimal linear codes have interesting applications in secret sharing [1,2,3,4,5] and secure two-party computation [6,7], and could be decoded with a minimum distance decoding method [8].

Up to now, there are two approaches to studying minimal linear codes. One is the algebraic method and the other is the geometric method. The algebraic method is based on the Hamming weights of the codewords. In [8], Ashikhmin and Barg gave a sufficient condition for a linear code to be minimal. Many minimal linear codes satisfying the condition $\frac{w_{min}}{w_{max}}>\frac{q-1}{q}$ are obtained from linear codes with few weights; for example [9,10]. Cohen et al. [7] provided an example to show that the condition $\frac{w_{min}}{w_{max}}>\frac{q-1}{q}$ is not necessary for a linear code to be minimal. Ding, Heng, and Zhou [11,12] derived a sufficient and necessary condition on all Hamming weights for a given linear code to be minimal.

When using the algebraic method to prove the minimality of a given linear code, one needs to know all the Hamming weights in the code, which is very difficult in general. Even if all the Hamming weights are known, it is hard to use the algebraic method to prove the minimality. In this paper, we will use the geometric approaches to study the minimality of some linear codes. Based on the geometric approaches (see [13,14,15]) it is easier to construct minimal linear codes or to prove the minimality of some linear codes (see [16,17,18,19,20,21]).

Sunflower in coding theory is a class of important subspace codes and can be used to construct linear codes, see [22]. Let *s* be the number of the elements in a sunflower. In [23], (Theorem 10), the authors proved that if s≥p+1, then the corresponding linear code over $F_{p}$ is minimal, where *p* is a prime number.

In this paper, we will use the approach used in [14] to consider the minimality of linear codes over $F_{q}$ constructed from sunflowers for all *s*. We obtain the following three results: (1) when s≥q+1, for any sunflower, the corresponding linear code is minimal; (2) when 2≤s≤3≤q, for any sunflower, the corresponding linear code is not minimal; (3) when 3<s≤q, for some sunflowers, the corresponding linear codes are minimal, wherea for some other sunflowers, the corresponding linear codes are not minimal.

This paper is organized as follows. In Section 2, we introduce some basic knowledge about sunflowers, Euclidean inner product, and minimal linear codes. In Section 3, we consider the linear codes constructed from sunflowers and discuss the minimality of these linear codes in three cases. In Section 4, we conclude this paper.

## 2. **Preliminaries**

### 2.1. Sunflower

Throughout this paper, let *k* and $t_{0}$ be two positive integers, $m=2k+t_{0}$ and $l=k+t_{0}$. Let $2\leq s\leq q^{k}+1$ be a positive integer, $T_{0}\leq F_{q}^{m}$ be a subspace of $F_{q}^{m}$, and $\dim T_{0}=t_{0}$. We denote $G_{q}\left(l,m\right)$ the set of *l*-dimensional vector subspaces of $F_{q}^{m}$. We define

$$\Phi =\{E_{i}\leq F_{q}^{m}:\;\dim E_{i}=l,\;E_{i}\cap E_{j}=T_{0},\;1\leq i\neq j\leq s\}.$$

Then, $\Phi \subseteq G_{q}\left(l,m\right)$ is a *sunflower* of $F_{q}^{m}$ and the space $T_{0}$ is called the center of the sunflower Φ.

**Lemma** **1.** *Let $\Phi \subseteq G_{q}\left(l,m\right)$ be a sunflower and $T_{0}$ the center of Φ. For any $E_{i}$, $E_{j}\in \Phi$ with 1≤i≠j≤s, we have $F_{q}^{m}=E_{i}+E_{j}$.*

**Proof.**  Since

$$\begin{array}{cc} \dim(E_{i}+E_{j}) & =\dim\left(E_{i}\right)+\dim\left(E_{j}\right)-\dim\left(E_{i}\cap E_{j}\right)\\ \; & =\dim\left(E_{i}\right)+\dim\left(E_{j}\right)-\dim\left(T_{0}\right)\\ \; & =l+l-t_{0}=m \end{array}$$

and $E_{i}+E_{j}\leq F_{q}^{m}$, we have $F_{q}^{m}=E_{i}+E_{j}$. □

**Lemma** **2.** *Let $\Phi \subseteq G_{q}\left(l,m\right)$ be a sunflower and $T_{0}$ the center of Φ. For any $E_{i}$, $E_{j}\in \Phi$ with 1≤i≠j≤s, we have $E_{i}^{⊥}\cap E_{j}^{⊥}=\left\{0\right\}$.*

**Proof.**  Assume that $z\in E_{i}^{⊥}\cap E_{j}^{⊥}$. It follows from Lemma 1 that $z\in {\left(F_{q}^{m}\right)}^{⊥}$, which implies z=0. □

### 2.2. Euclidean Inner Product

Let *m* be a positive integer. For $x=(x_{1},x_{2},\ldots ,x_{m})$, $y=\left(y_{1},y_{2},\ldots ,y_{m}\right)\in \;F_{q}^{m}$, the *Euclidean inner product* of x and y is given by

$$<x,y>:=xy^{T}=\sum_{i=1}^{m}x_{i}y_{i}.$$

For any $S\subseteq F_{q}^{m}$, we define

$$\mathrm{Span}\left(S\right):=\left\{\sum_{i=1}^{r}\lambda_{i}s_{i}\;|\;r\in N,s_{i}\in S,\lambda_{i}\in F_{q}\right\},$$

$$S^{⊥}:=\left\{v\in F_{q}^{m}\;|\;{\mathrm{vs}}^{T}=0,\;\mathrm{for}\;\mathrm{any}\;s\in S\right\}.$$

Then, Span(S) and $S^{⊥}$ are vector spaces over $F_{q}$ and

(1) $$\dim(\mathrm{Span}\left(S\right))+\dim\left(S^{⊥}\right)=m.$$

### 2.3. Minimal Linear Codes

All linear codes can be constructed by the following way. Let m≤n be two positive integers. Let $G:=[d_{1},\ldots ,d_{n}]$ be an m×n matrix over $F_{q}$ and $D:=\{d_{1},\ldots ,d_{n}\}$ be a multiset. Let r(D)=r(G) denote the rank of *G*, which is equal to the dimension of the vector space Span(D) over $F_{q}$. Let

$$C\left(D\right):=\left\{c(x)=xG=\left({\mathrm{xd}}_{1}^{T},\ldots ,{\mathrm{xd}}_{n}^{T}\right),x\in F_{q}^{m}\right\}.$$

Then, C(D) is an [n, ${r\left(D\right)]}_{q}$ linear code with generator matrix *G*. We always study the minimality of C(D) by considering some appropriate multisets *D*.

To present the sufficient and necessary condition for minimal linear codes in [14], some concepts are needed. For any $y\in F_{q}^{m}$, we define

$$H\left(y\right):=y^{⊥}=\left\{x\in F_{q}^{m}|{\mathrm{xy}}^{T}=0\right\},$$

$$H\left(y,D\right):=D\cap H\left(y\right)=\left\{x\in D|{\mathrm{xy}}^{T}=0\right\},$$

V(y,D):=Span(H(y,D)).

It is obvious that H(y,D)⊆V(y,D)⊆H(y).

**Proposition** **1** ([14]). *For any $x,y\in F_{q}^{m},\;c\left(x\right)⪯c\left(y\right)$ if and only if H(y,D)⊆H(x,D).*

Let $y\in F_{q}^{m}\setminus \left\{0\right\}$. The following lemma gives a sufficient and necessary condition for the codeword c(y)∈C(D) to be minimal.

**Lemma** **3** ([14] (Theorem 3.1)). *Let $y\in F_{q}^{m}\setminus \left\{0\right\}$. Then, the following three conditions are equivalent:*

(1) *c(y) is minimal in C(D);*
(2) *dimV(y,D)=m−1;*
(3) *V(y,D)=H(y).*

The following lemma gives a sufficient and necessary condition for linear codes over $F_{q}$ to be minimal.

**Lemma** **4** ([14] (Theorem 3.2)). *The following three conditions are equivalent:*

(1) *C(D) is minimal;*
(2) *for any $y\in F_{q}^{m}\setminus \left\{0\right\}$, dimV(y,D)=m−1;*
(3) *for any $y\in F_{q}^{m}\setminus \left\{0\right\}$, V(y,D)=H(y).*

By the following lemma, we can obtain infinity of many minimal linear codes from any known minimal linear codes.

**Lemma** **5** ([14] (Proposition 4.1)). *Let $D_{1}\subseteq D_{2}$ be two multisets with elements in $F_{q}^{m}$ and $r\left(D_{1}\right)=r\left(D_{2}\right)=m$. If $C(D_{1})$ is minimal, then $C(D_{2})$ is minimal.*

The following corollary is trivial.

**Corollary** **1.** *Let $D_{1}\subseteq D_{2}$ be two multisets with elements in $F_{q}^{m}$ and $r\left(D_{1}\right)=r\left(D_{2}\right)=m$. If $C(D_{2})$ is not minimal, then $C(D_{1})$ is not minimal.*

In the following section, we will use the above lemmas to consider the minimality of linear codes constructed from sunflowers.

## 3. The Minimality of Linear Codes Constructed from Sunflowers

In this section, we consider the linear codes constructed from sunflowers and discuss the minimality of these linear codes.

Let

$$\Phi =\{E_{i}\leq F_{q}^{m}:\;\dim E_{i}=l,E_{i}\cap E_{j}=T_{0},1\leq i\neq j\leq s\}.$$

be a sunflower of $F_{q}^{m}$ and $T_{0}$ the center of Φ.

Let

(2) $$D:=\left(\overset{s}{\underset{i=1}{⋃}}E_{i}\right)\setminus T_{0}=\overset{s}{\underset{i=1}{⋃}}\left(E_{i}\setminus T_{0}\right).$$

It is easy to see that C(D) is a ${\left[s\left(q^{l}-q^{t_{0}}\right),m\right]}_{q}$ linear code.

The following lemmas are important in the proofs of this section.

**Lemma** **6** ([24] (Lemma 3.1)). *For all $y\in F_{q}^{m}\setminus \left\{0\right\}$, $E\leq F_{q}^{m}$ and dim(E)=r, we have H(y,E)=V(y,E) and*

$$\dim V\left(y,E\right)=\left\{\begin{array}{ccc} & r, & if\;y\in E^{⊥};\\ & r-1, & if\;y\notin E^{⊥}. \end{array}\right.$$

By linear algebra, we can obtain the following lemma.

**Lemma** **7.** *Let $y\in F_{q}^{m}\setminus \left\{0\right\}$. If for any $E_{i}\in \Phi$, $y\notin {E_{i}}^{⊥}$, 1≤i≤s. For any $E_{i_{0}}$, $E_{j_{0}}\in \Phi$, $E_{i_{0}}\neq E_{j_{0}}$, let $D_{1}=\left(E_{i_{0}}\cup E_{j_{0}}\right)\setminus T_{0}$. We have*

$$\mathrm{rank}H\left(y,D_{1}\right)=\left\{\begin{array}{ccc} & m-2, & if\;y\in {T_{0}}^{⊥};\\ & m-1, & if\;y\notin {T_{0}}^{⊥}. \end{array}\right.$$

**Proof.**  Since $y\notin E_{i}^{⊥}$, it follows from Lemma 6 that $\dim H\left(y,E_{i_{0}}\right)=\dim H\left(y,E_{j_{0}}\right)=l-1$. Note that $H\left(y,T_{0}\right)\leq H\left(y,E_{i_{0}}\right)$ and $H\left(y,T_{0}\right)\leq H\left(y,E_{j_{0}}\right).$

If $y\in T_{0}^{⊥}$, then $H\left(y,T_{0}\right)=T_{0}$. Suppose that

$$\begin{array}{c} H\left(y,T_{0}\right)=T_{0}=\mathrm{Span}\left\{\gamma_{1},\gamma_{2},\ldots ,\gamma_{t_{0}}\right\},\\ H\left(y,E_{i_{0}}\right)=\mathrm{Span}\left\{\alpha_{1},\alpha_{2},\ldots ,\alpha_{k-1},\gamma_{1},\gamma_{2},\ldots ,\gamma_{t_{0}}\right\},\\ H\left(y,E_{j_{0}}\right)=\mathrm{Span}\left\{\beta_{1},\beta_{2},\ldots ,\beta_{k-1},\gamma_{1},\gamma_{2},\ldots ,\gamma_{t_{0}}\right\}. \end{array}$$

Then, we have

$$\begin{array}{cc} & H\left(y,E_{i_{0}}\right)\setminus T_{0}⊇\left\{\alpha_{1},\alpha_{2}\ldots ,\alpha_{k-1},\alpha_{1}+\gamma_{1},\alpha_{1}+\gamma_{2},\ldots ,\alpha_{1}+\gamma_{t_{0}}\right\},\\ & H\left(y,E_{j_{0}}\right)\setminus T_{0}⊇\left\{\beta_{1},\beta_{2},\ldots ,\beta_{k-1},\beta_{1}+\gamma_{1},\beta_{1}+\gamma_{2},\ldots ,\beta_{1}+\gamma_{t_{0}}\right\}. \end{array}$$

Since $H\left(y,D_{1}\right)=\left(H\left(y,E_{i_{0}}\right)\cup H\left(y,E_{j_{0}}\right)\right)\setminus T_{0}$, the above equations lead to

$$H\left(y,D_{1}\right)⊇\left\{\alpha_{1},\alpha_{2},\ldots ,\alpha_{k-1},\beta_{1},\beta_{2},\ldots ,\beta_{k-1},\alpha_{1}+\gamma_{1},\alpha_{1}+\gamma_{2},\ldots ,\alpha_{1}+\gamma_{t_{0}}\right\},$$

i.e., $\mathrm{rank}H(y,D_{1})=m-2$.

If $y\notin T_{0}^{⊥}$, then $\dim H\left(y,T_{0}\right)=t_{0}-1$ by Lemma 6. Suppose that

$$\begin{array}{c} H\left(y,T_{0}\right)=\mathrm{Span}\left\{\gamma_{1},\gamma_{2},\ldots ,\gamma_{t_{0}-1}\right\},\\ T_{0}=\mathrm{Span}\left\{\gamma_{1},\gamma_{2},\ldots ,\gamma_{t_{0}-1},\gamma_{t_{0}}\right\},\\ H\left(y,E_{i_{0}}\right)=\mathrm{Span}\left\{\alpha_{1},\alpha_{2},\ldots ,\alpha_{k},\gamma_{1},\gamma_{2},\ldots ,\gamma_{t_{0}-1}\right\},\\ H\left(y,E_{j_{0}}\right)=\mathrm{Span}\left\{\beta_{1},\beta_{2},\ldots ,\beta_{k},\gamma_{1},\gamma_{2},\ldots ,\gamma_{t_{0}-1}\right\}. \end{array}$$

Then, we have

$$\begin{array}{cc} & H\left(y,E_{i_{0}}\right)\setminus T_{0}⊇\left\{\alpha_{1},\alpha_{2}\ldots ,\alpha_{k},\alpha_{1}+\gamma_{1},\alpha_{1}+\gamma_{2},\ldots ,\alpha_{1}+\gamma_{t_{0}-1}\right\},\\ & H\left(y,E_{j_{0}}\right)\setminus T_{0}⊇\left\{\beta_{1},\beta_{2},\ldots ,\beta_{k},\beta_{1}+\gamma_{1},\beta_{1}+\gamma_{2},\ldots ,\beta_{1}+\gamma_{t_{0}-1}\right\}. \end{array}$$

Since $H\left(y,D_{1}\right)=\left(H\left(y,E_{i_{0}}\right)\cup H\left(y,E_{j_{0}}\right)\right)\setminus T_{0}$, the above equations yield

$$H\left(y,D_{1}\right)⊇\left\{\alpha_{1},\alpha_{2},\ldots ,\alpha_{k},\beta_{1},\beta_{2},\ldots ,\beta_{k},\alpha_{1}+\gamma_{1},\alpha_{1}+\gamma_{2},\ldots ,\alpha_{1}+\gamma_{t_{0}-1}\right\},$$

i.e., $\mathrm{rank}H(y,D_{1})=m-1$. The proof is completed. □

Now, we consider the minimality of C(D) in three cases. First, when s≥q+1, we have

**Theorem** **1.** *Let $\Phi =\{E_{1},\ldots ,E_{s}\}$ be a sunflower of $F_{q}^{m}$ with center $T_{0}$ of dimension $t_{0}$. If s≥q+1, then C(D) is an ${\left[s\left(q^{l}-q^{t_{0}}\right),m\right]}_{q}$ minimal linear code.*

**Proof.**  According to Lemma 4, we only need to prove that for any $y\in F_{q}^{m}\setminus \left\{0\right\}$, dimV(y,D)=m−1. By (2), we obtain

(3) $$H\left(y,D\right)=D\cap H\left(y\right)=\overset{s}{\underset{i=1}{⋃}}\left(H\left(y,E_{i}\right)\setminus T_{0}\right).$$

There are three cases:

(1) If there exists $E_{i_{0}}\in \Phi$ such that $y\in E_{i_{0}}^{⊥}$, then we have $\dim H(y,E_{i_{0}})=l$ from Lemma 6. According to Lemma 2, for any $E_{j_{0}}\in \Phi$ with $E_{j_{0}}\neq E_{i_{0}}$, we have $y\notin E_{j_{0}}^{⊥}.$ Then, it follows from Lemma 6 that $\dim H(y,E_{j_{0}})=l-1.$ Since $y\in E_{i_{0}}^{⊥}\subseteq T_{0}^{⊥}$, we have $H\left(y,T_{0}\right)=T_{0}$. We set

$$H\left(y,T_{0}\right)=T_{0}=\mathrm{Span}\left\{\gamma_{1},\gamma_{2},\ldots ,\gamma_{t_{0}}\right\}.$$

When k=1, we set

$$H\left(y,E_{i_{0}}\right)=\mathrm{Span}\left\{\alpha_{1},\gamma_{1},\gamma_{2},\ldots ,\gamma_{t_{0}}\right\}.$$

By (3), we have $H\left(y,D\right)⊇\left\{\alpha_{1},\alpha_{1}+\gamma_{1},\alpha_{1}+\gamma_{2},\ldots ,\alpha_{1}+\gamma_{t_{0}}\right\}$, and so dimV(y,D)=m−1.

When k>1, we set

$$H\left(y,E_{i_{0}}\right)=\mathrm{Span}\left\{\alpha_{1},\alpha_{2},\ldots ,\alpha_{k},\gamma_{1},\gamma_{2},\ldots ,\gamma_{t_{0}}\right\},$$

and

$$H\left(y,E_{j_{0}}\right)=\mathrm{Span}\left\{\beta_{1},\beta_{2},\ldots ,\beta_{k-1},\gamma_{1},\gamma_{2},\ldots ,\gamma_{t_{0}}\right\}.$$

By (3), we have

$$H\left(y,D\right)⊇\left\{\alpha_{1},\alpha_{2},\ldots ,\alpha_{k},\beta_{1},\beta_{2},\ldots ,\beta_{k-1},\alpha_{1}+\gamma_{1},\alpha_{1}+\gamma_{2},\ldots ,\alpha_{1}+\gamma_{t_{0}}\right\}.$$

Since

$$\mathrm{rank}\{\alpha_{1},\alpha_{2},\ldots ,\alpha_{k},\beta_{1},\beta_{2},\ldots ,\beta_{k-1},\alpha_{1}+\gamma_{1},\alpha_{1}+\gamma_{2},\ldots ,\alpha_{1}+\gamma_{t_{0}}\}=m-1,$$

it is easy to obtain dimV(y,D)=m−1.

(2) If for any $E_{i}\in \Phi$, 1≤i≤s, we have $y\notin E_{i}^{⊥}$ and $y\notin T_{0}^{⊥}$, then $\dim H(y,E_{i_{0}})=$ dim$H(y,E_{j_{0}})=l-1$ for any $E_{i_{0}},E_{j_{0}}\in \Phi$ with $E_{i_{0}}\neq E_{j_{0}}$. Since $y\notin T_{0}^{⊥}$, $\dim H\left(y,T_{0}\right)=t_{0}-1.$ We set

$$H\left(y,T_{0}\right)=\mathrm{Span}\left\{\gamma_{1},\gamma_{2},\ldots ,\gamma_{t_{0}-1}\right\},\;T_{0}=\mathrm{Span}\left\{\gamma_{1},\gamma_{2},\ldots ,\gamma_{t_{0}}\right\}.$$

When k=1, we set

$$H\left(y,E_{i_{0}}\right)=\mathrm{Span}\left\{\alpha_{1},\gamma_{1},\gamma_{2},\ldots ,\gamma_{t_{0}-1}\right\},\;H\left(y,E_{j_{0}}\right)=\mathrm{Span}\left\{\beta_{1},\gamma_{1},\gamma_{2},\ldots ,\gamma_{t_{0}-1}\right\}.$$

Then,

$$H\left(y,D\right)⊇\left\{\alpha_{1},\beta_{1},\alpha_{1}+\gamma_{1},\alpha_{1}+\gamma_{2},\ldots ,\alpha_{1}+\gamma_{t_{0}-1}\right\}.$$

Since

$$\mathrm{rank}\{\alpha_{1},\beta_{1},\alpha_{1}+\gamma_{1},\alpha_{1}+\gamma_{2},\ldots ,\alpha_{1}+\gamma_{t_{0}-1}\}=m-1,$$

it is easy to obtain dimV(y,D)=m−1.

When k>1, let $D_{1}=\left(E_{i_{0}}\cup E_{j_{0}}\right)\setminus T_{0}$. By Lemma 7, we have rank$(H\left(y,D_{1}\right))=m-1,$ thus dimV(y,D)=m−1.

(3) If for any $E_{i}\in \Phi$, 1≤i≤s, we have $y\notin E_{i}^{⊥}$ and $y\in T_{0}^{⊥}$; then, it follows from Lemma 6 that $\dim H(y,E_{i})=l-1$ and $\dim H\left(y,T_{0}\right)=t_{0}.$

When k=1, we obtain $\dim T_{0}^{⊥}$=2 and $\dim E_{i}^{⊥}$=1, 1≤i≤s, then $E_{i}^{⊥}$ is the one-dimensional subspace of $T_{0}^{⊥}$. There are q+1 one dimensional subspace of $T_{0}^{⊥}$, since s≥q+1, we obtain s=q+1. By Lemma 2, for any $E_{i}$, $E_{j}\in \Phi$, $E_{i}\neq E_{j}$, we have $E_{i}^{⊥}\cap E_{j}^{⊥}=\left\{0\right\}$. Thus,

(4) $$T_{0}^{⊥}=\overset{s}{\underset{i=1}{⋃}}E_{i}^{⊥}.$$

Since $y\in T_{0}^{⊥}$, by (4), there exists $E_{j}\in \Phi ,$ such that $y\in E_{j}^{⊥},$ a contradiction. So k≠1.

When k>1, we have $\dim H(y,E_{1})$=dim$H(y,E_{2})=l-1.$ Let $D_{1}=\left(E_{1}\cup E_{2}\right)\setminus T_{0}$. By Lemma 7, we have rank$(H\left(y,D_{1}\right))=m-2.$ We set

$$T_{0}=H\left(y,T_{0}\right)=\mathrm{Span}\left\{\gamma_{1},\gamma_{2},\ldots ,\gamma_{t_{0}}\right\}.$$

$$E_{1}=\mathrm{Span}\left\{\alpha_{1},\alpha_{2},\ldots ,\alpha_{k-1},\alpha_{k},\gamma_{1},\gamma_{2},\ldots ,\gamma_{t_{0}}\right\},\;H\left(y,E_{1}\right)=\mathrm{Span}\left\{\alpha_{1},\alpha_{2},\ldots ,\alpha_{k-1},\gamma_{1},\gamma_{2},\ldots ,\gamma_{t_{0}}\right\}.$$

$$E_{2}=\mathrm{Span}\left\{\beta_{1},\beta_{2},\ldots ,\beta_{k-1},\beta_{k},\gamma_{1},\gamma_{2},\ldots ,\gamma_{t_{0}}\right\},\;H\left(y,E_{2}\right)=\mathrm{Span}\left\{\beta_{1},\beta_{2},\ldots ,\beta_{k-1},\gamma_{1},\gamma_{2},\ldots ,\gamma_{t_{0}}\right\}.$$

Let

$$B=\{\alpha_{1},\alpha_{2},\ldots ,\alpha_{k-1},\beta_{1},\beta_{2},\ldots ,\beta_{k-1},\alpha_{1}+\gamma_{1},\alpha_{1}+\gamma_{2},\ldots ,\alpha_{1}+\gamma_{t_{0}}\}.$$

Then, rankB=m−2 and B⊆H(y,D). Let $V=F_{q}^{m}$, W=Span(B) and $\bar{V}=V/W$ the quotient space of *V* over *W*. We have $\dim\bar{V}=2$ and V=Span$\left\{\bar{\alpha_{k}},\bar{\beta_{k}}\right\}$. Let π be the standard map from *V* to $\bar{V}$. For any $E_{i}\in \Phi$, 1≤i≤s, $\pi (E_{i})$ is a subspace of $\bar{V}$. It is easily seen that $\dim\pi (E_{i})=1$ or 2. There are the following two cases.

(i) If there exists $E_{i_{0}}\in \Phi$ such that $\dim\pi (E_{i_{0}})=2$, then $\pi \left(E_{i_{0}}\right)=\bar{V}$. There must exist $\alpha \in E_{i_{0}}$ such that

$$\pi \left(\alpha \right)=\bar{\alpha_{k}-b\beta_{k}},\;\mathrm{where}\;b=\left(\alpha_{k}y^{T}\right)/\left(\beta_{k}y^{T}\right).$$

So, $\alpha =\alpha_{k}-b\beta_{k}+w$, where w∈W. It is simply checked that $\alpha \notin T_{0}$, α∈H(y) and α∉W. We obtain

(B∪{α})⊆H(y,D),rank(B∪{α})=m−1.

Thus, dimV(y,D)=m−1.

(ii) If for any $E_{i}\in \Phi$ we have $\dim\pi (E_{i})=1$, combining that $V=E_{i}+E_{j}$ for any $E_{i}$, $E_{j}\in \Phi$ with $E_{i}\neq E_{j}$ in accordance with Lemma 1, we have

$$\bar{V}=\pi \left(V\right)=\pi \left(E_{i}\right)+\pi \left(E_{j}\right)\;\mathrm{and}\;\pi \left(E_{i}\right)\neq \pi \left(E_{j}\right).$$

Since $\bar{V}$ has only q+1 one-dimensional subspace and s≥q+1, we have s=q+1 and $\bar{V}=\overset{s}{\underset{i=1}{⋃}}\pi \left(E_{i}\right)$. There must exist $E_{j_{0}}\in \Phi$ such that

$$\pi \left(E_{j_{0}}\right)=\mathrm{Span}\left\{\bar{\alpha_{k}-b\beta_{k}}\right\},\;\mathrm{where}\;b=\left(\alpha_{k}y^{T}\right)/\left(\beta_{k}y^{T}\right).$$

Hence, there exists $\alpha =\alpha_{k}-b\beta_{k}+w\in E_{j_{0}}$, where w∈W, such that $\pi \left(\alpha \right)=\bar{\alpha_{k}-b\beta_{k}}$. One can easily deduce that $\alpha \notin T_{0}$, α∈H(y) and α∉W. We obtain

(B∪{α})⊆H(y,D),rank(B∪{α})=m−1.

Thus, dimV((y),D)=m−1.

In conclusion, for any $y\in F_{q}^{m}\setminus \left\{0\right\},$ we have dimV(y,D)=m−1, so C(D) is a minimal linear code. □

**Remark** **1.** *In Theorem 1, if q=p is a prime number, then it becomes [23] (Theorem 10). So Theorem 1 is a generalization of [23] (Theorem 10). Our method is different from theirs. When s≤q, our method also can be used to study the minimality of the linear codes, whereas theirs can not.*

**Example** **1.** *Let $e_{1},\ldots ,e_{m}$ be the standard basis of $F_{q}^{m}$. Let*

(5) $$T_{0}^{\prime }=\mathrm{Span}\left(\left\{e_{2k+1},e_{2k+2},\ldots ,e_{m}\right\}\right)=\left\{\left(0,0,t\right)|t\in F_{q}^{t_{0}}\right\}.$$

 *For any $b\in F_{q}$, we define*

(6) $$E_{b}=\mathrm{Span}\left\{e_{1}+be_{k+1},e_{2}+be_{k+2},\ldots ,e_{k}+be_{2k},e_{2k+1},\ldots ,e_{m}\right\}.$$

 *Suppose that*

$$\Phi =\left\{E_{b}|b\in F_{q}\right\}\cup \mathrm{Span}\left\{e_{k+1},e_{k+2},\ldots ,e_{2k},e_{2k+1},\ldots ,e_{m}\right\}$$

 *and* 

$$D^{\prime }=\underset{E_{i}\in \Phi }{⋃}\left(E_{i}\setminus T_{0}^{\prime }\right).$$

 *It is easy to see that Φ is a sunflower of $F_{q}^{m}$ with center $T_{0}^{\prime }$ and s=q+1. Here, we take q=4,k=3, and $t_{0}=1$. With the help of Magma, we verify that the code $C(D^{\prime })$ is a minimal ${\left[1260,7\right]}_{4}$ linear code with minimum distance 768, and*

$$\frac{w_{\min}}{w_{\max}}=\frac{4}{5}>\frac{3}{4}.$$

Now, we consider the minimality of C(D) when 2≤s≤3≤q. If s=3, we have

**Theorem** **2.** *Let $\Phi =\{E_{1},\ldots ,E_{s}\}$ be a sunflower of $F_{q}^{m}$ with center $T_{0}$ of dimension $t_{0}$. If s=3≤q, then C(D) is not minimal.*

**Proof.**  To prove C(D) is not minimal, by Lemma 4, we only need to prove there exists $y_{0}\in F_{q}^{m}\setminus \left\{0\right\}$ such that $\dim V(y_{0},D)\leq m-2.$

When s=3, $\Phi =\{E_{1},E_{2},E_{3}\}$. By Lemma 2 we know $E_{1}^{⊥}\cap E_{2}^{⊥}=\left\{0\right\}$. Then, for any $y_{1}\in E_{2}^{⊥}\setminus \left\{0\right\}$, we have $y_{1}\notin E_{1}^{⊥}$ and $y_{1}\in T_{0}^{⊥}$. Thus, $\dim H(y_{1},E_{1})=l-1$, $\dim H(y_{1},E_{2})=l$, and $\dim H\left(y_{1},T_{0}\right)=t_{0}$. We set

$$T_{0}=H\left(y_{1},T_{0}\right)=\mathrm{Span}\left\{\gamma_{1},\gamma_{2},\ldots ,\gamma_{t_{0}}\right\},$$

$$E_{1}=\mathrm{Span}\left\{\alpha_{1},\alpha_{2},\ldots ,\alpha_{k-1},\alpha_{k},\gamma_{1},\gamma_{2},\ldots ,\gamma_{t_{0}}\right\},\;H\left(y_{1},E_{1}\right)=\mathrm{Span}\left\{\alpha_{1},\alpha_{2},\ldots ,\alpha_{k-1},\gamma_{1},\gamma_{2},\ldots ,\gamma_{t_{0}}\right\},$$

$$E_{2}=H\left(y_{1},E_{2}\right)=\mathrm{Span}\left\{\beta_{1},\beta_{2},\ldots ,\beta_{k},\gamma_{1},\gamma_{2},\ldots ,\gamma_{t_{0}}\right\},$$

where $\alpha_{k}y_{1}^{T}=1$. Let

$$E_{1}^{\prime }=\mathrm{Span}\left\{\alpha_{1},\alpha_{2},\ldots ,\alpha_{k}\right\},\;E_{2}^{\prime }=\mathrm{Span}\left\{\beta_{1},\beta_{2},\ldots ,\beta_{k}\right\},$$

we have $F_{q}^{m}=E_{1}^{\prime }⊕E_{2}^{\prime }⊕T_{0}.$ For any $\eta \in E_{3},$ there exist unique $\alpha \in E_{1}^{\prime }$, $\beta \in E_{2}^{\prime },\gamma \in T_{0}$, such that η=α+β+γ. Since $\alpha +\beta =\eta -\gamma \in E_{3}$, for any $\alpha \in E_{1}^{\prime }$, there exists unique $\beta \in E_{2}^{\prime }$ such that $\alpha +\beta \in E_{3}$. Let φ be a map from $E_{1}^{\prime }$ to $E_{2}^{\prime }$ satisfying φ(α)=β. We can see φ is an isomorphism from $E_{1}^{\prime }$ to $E_{2}^{\prime }$ and

$$E_{3}=\left\{x+\varphi \left(x\right)|x\in E_{1}^{\prime }\right\}⊕T_{0}=E_{3}^{\prime }⊕T_{0}.$$

Since $y_{1}\notin E_{1}^{⊥}$, $y_{1}\in T_{0}^{⊥}$, and $E_{1}=E_{1}^{\prime }⊕T_{0}$, we have $y_{1}\notin {\left(E_{1}^{\prime }\right)}^{⊥}$, $\dim H(y_{1},E_{1}^{\prime })=k-1,$$\dim\varphi (H\left(y_{1},E_{1}^{\prime }\right))=k-1$, and $\dim\varphi {\left(H\left(y_{1},E_{1}^{\prime }\right)\right)}^{⊥}=m-\left(k-1\right)=k+t_{0}+1.$ Thus,

$$\begin{array}{cc} & \dim(\varphi {\left(H\left(y_{1},E_{1}^{\prime }\right)\right)}^{⊥}\cap E_{1}^{⊥})\\ = & \dim\varphi {\left(H\left(y_{1},E_{1}^{\prime }\right)\right)}^{⊥}+\dim\left(E_{1}^{⊥}\right)-\dim\left(\varphi {\left(H\left(y_{1},E_{1}^{\prime }\right)\right)}^{⊥}+E_{1}^{⊥}\right)\\ \geq & k+t_{0}+1+k-m=1. \end{array}$$

Since q≥3, there exists $y_{2}\in \left(\varphi {\left(H\left(y_{1},E_{1}^{\prime }\right)\right)}^{⊥}\cap E_{1}^{⊥}\right)\setminus \left\{0\right\}$ such that $\varphi \left(\alpha_{k}\right)y_{2}^{T}\neq -1$. It is easy to see $y_{2}\notin E_{2}^{⊥}$ and $y_{2}\in T_{0}^{⊥}.$ Let $y_{0}=y_{1}+y_{2}$, we obtain $y_{0}\notin E_{1}^{⊥}$, $y_{0}\notin E_{2}^{⊥}$, and $y_{0}\in T_{0}^{⊥}$. Since $\alpha_{k}+\varphi \left(\alpha_{k}\right)\in E_{3}$ and

$$\begin{array}{cc} \left(\alpha_{k}+\varphi \left(\alpha_{k}\right)\right)y_{0}^{T} & =\left(\alpha_{k}+\varphi \left(\alpha_{k}\right)\right){\left(y_{1}+y_{2}\right)}^{T}\\ & =\alpha_{k}y_{1}^{T}+\alpha_{k}y_{2}^{T}+\varphi \left(\alpha_{k}\right)y_{1}^{T}+\varphi \left(\alpha_{k}\right)y_{2}^{T}\\ & =1+0+0+\varphi \left(\alpha_{k}\right)y_{2}^{T}\neq 0, \end{array}$$

we obtain $y_{0}\notin E_{3}^{⊥}.$ Thus, $y_{0}\notin E_{i}^{⊥},\;1\leq i\leq 3$ and $\dim H(y_{0},E_{i})=l-1.$

(1) When k=1, $\dim H\left(y_{0},E_{i}\right)=t_{0}$, since $T_{0}\leq H\left(y_{0},E_{i}\right)$, we have $T_{0}=H\left(y_{0},E_{i}\right)$. Thus,

$$H\left(y_{0},D\right)=\overset{3}{\underset{i=1}{⋃}}\left(H\left(y,E_{i}\right)\setminus T_{0}\right)=\emptyset .$$

Thus, C(D) is not minimal.

(2) When k>1, since $E_{i}=E_{i}^{\prime }⊕T_{0}$, we have $y_{0}\notin {\left(E_{i}^{\prime }\right)}^{⊥}$ and $\dim H(y_{0},E_{i}^{\prime })=k-1.$ Thus,

$$H\left(y_{0},E_{i}\right)=H\left(y_{0},E_{i}^{\prime }\right)⊕T_{0},\;1\leq i\leq 3.$$

By Lemma 6, it is easily verified that

$$H\left(y_{0},E_{1}^{\prime }\right)=H\left(y_{1},E_{1}^{\prime }\right),$$

$$H\left(y_{0},E_{2}^{\prime }\right)=H\left(y_{2},E_{2}^{\prime }\right)=\varphi \left(H\left(y_{1},E_{1}^{\prime }\right)\right)=\varphi \left(H\left(y_{0},E_{1}^{\prime }\right)\right),$$

$$H\left(y_{0},E_{3}^{\prime }\right)=\left\{x+\varphi \left(x\right)|x\in H\left(y_{0},E_{1}^{\prime }\right)\right\}\subseteq \mathrm{Span}\left(H\left(y_{0},E_{1}^{\prime }\right)\cup H\left(y_{0},E_{2}^{\prime }\right)\right).$$

Then, $\dim V(y_{0},D)=m-2$. By Lemma 4, we have that $c(y_{0})$ is not minimal. □

Combining Theorem 2 and Corollary 1, we have

**Corollary** **2.** *Let $\Phi =\{E_{1},\ldots ,E_{s}\}$ be a partial spread of $F_{q}^{m}$. If 2≤s≤3≤q, then C(D) is not minimal.*

Now, we consider the minimality of C(D) when 4≤s≤q. We recall from (5) that

$$T_{0}^{\prime }=\mathrm{Span}\left(\left\{e_{2k+1},e_{2k+2},\ldots ,e_{m}\right\}\right)=\left\{\left(0,0,t\right)|t\in F_{q}^{t_{0}}\right\}.$$

We will show that some sunflowers Φ with center $T_{0}^{\prime }$, C(D) are minimal, whereas some other sunflowers Φ with center $T_{0}^{\prime }$, C(D) are not minimal.

First, we construct some sunflowers Φ such that C(D) are minimal. Let k≥2, f(x) be an irreducible polynomial in $F_{q}\left[x\right]$ of degree *k* and $M\in F_{q}^{k\times k}$ be a matrix with characteristic polynomial f(x). We define

(7) $$\begin{array}{cc} \; & E_{1}=\left\{\left(x,0,t\right)|x\in F_{q}^{k},t\in F_{q}^{t_{0}}\right\},E_{2}=\left\{\left(0,x,t\right)|x\in F_{q}^{k},t\in F_{q}^{t_{0}}\right\},\\ \; & E_{3}=\left\{\left(x,x,t\right)|x\in F_{q}^{k},t\in F_{q}^{t_{0}}\right\},E_{4}=\left\{\left(x,xM,t\right)|x\in F_{q}^{k},t\in F_{q}^{t_{0}}\right\}, \end{array}$$

and

(8) $$\Phi =\{E_{1},E_{2},E_{3},E_{4}\}.$$

We can see Φ is a sunflower with center $T_{0}^{\prime }$.

**Theorem** **3.** *For the sunflower *Φ* defined in (8), the linear code C(D) is minimal.*

**Proof.**  According to Lemma 4, we only need to prove that for any $y\in F_{q}^{m}\setminus \left\{0\right\}$, dimV(y,D)=m−1. There are three cases:

(1) If there exists $E_{i_{0}}\in \Phi$ such that $y\in E_{i_{0}}^{⊥}$, the proof is similar as that in Theorem 1 (1).
(2) If for any $E_{i}\in \Phi$, 1≤i≤s, we have $y\notin E_{i}^{⊥}$ and $y\notin T_{0}^{\prime ⊥}$, then the proof is similar to that in Theorem 1 (2).
(3) If for any $E_{i}\in \Phi$, 1≤i≤s, we have $y\notin E_{i}^{⊥}$ and $y\in T_{0}^{\prime ⊥}$, the proof is as follows. Let $y=(y_{1},y_{2},y_{3})$ where $y_{1},y_{2}\in F_{q}^{k},y_{3}\in F_{q}^{t_{0}}$. Next, we define two linear transformations φ, ψ from $F_{q}^{k}$ to $F_{q}^{k}$:
  (9) $$\varphi \left(x\right)=x,\psi \left(x\right)=xM,x\in F_{q}^{k}.$$
  Then,
  (10) $$\begin{array}{cc} \; & E_{3}=\left\{\left(x,\varphi (x),t\right)|x\in F_{q}^{k},t\in F_{q}^{t_{0}}\right\},E_{4}=\left\{\left(x,\varphi (x),t\right)|x\in F_{q}^{k},t\in F_{q}^{t_{0}}\right\}. \end{array}$$
  Let
  (11) $$\begin{array}{cc} \; & E_{1}^{\prime }=\left\{\left(x,0,0\right)|x\in F_{q}^{k}\right\},E_{2}^{\prime }=\left\{\left(0,x,0\right)|x\in F_{q}^{k}\right\},\\ \; & E_{3}^{\prime }=\left\{\left(x,\varphi \left(x\right),0\right)|x\in F_{q}^{k}\right\},E_{4}^{\prime }=\left\{\left(x,\psi \left(x\right),0\right)|x\in F_{q}^{k}\right\}. \end{array}$$
  It is easy to verify that
  $$E_{i}=E_{i}^{\prime }⊕T_{0}^{\prime },\;1\leq i\leq 4.$$
  Let
  (12) $$\begin{array}{cc} S: & =\mathrm{Span}\{H\left(y,E_{1}\right)\cup H\left(y,E_{2}\right)\}\\ \; & =\mathrm{Span}\{\left\{H\left(y,E_{1}\right)\cup H\left(y,E_{2}\right)\right\}\setminus T_{0}^{\prime }\}\\ \; & =\left\{\left(\alpha ,\beta ,0\right)|\alpha \in H\left(y,E_{1}\right),\beta \in H\left(y,E_{2}\right)\right\}⊕\left\{\left(0,0,t\right)|t\in F_{q}^{t_{0}}\right\}\\ \; & =S^{\prime }⊕T_{0}^{\prime }. \end{array}$$
  By Lemma 7, we have dim.

Now, we prove $H(y,E_{3})⊈S$ or $H(y,E_{4})⊈S.$ If not, assume that $H(y,E_{3})\subseteq S$ and $H(y,E_{4})\subseteq S$. By $H(y,E_{3})\subseteq S$, it is obvious that $H\left(y,E_{3}^{\prime }\right)\subseteq S^{\prime }$. Since $y\notin E_{3}^{⊥}$ and $y\in T_{0}^{\prime ⊥}$, we have $y\notin E_{3}^{\prime ⊥}$, and then $\dim H(y,E_{3}^{\prime })=k-1.$ There exists $\alpha_{1},\ldots ,\alpha_{k-1}\in H\left(y_{1}\right)$, $\beta_{1},\ldots ,\beta_{k-1}\in H\left(y_{2}\right)$ such that $\left(\alpha_{1},\beta_{1},0\right),\ldots ,\left(\alpha_{k-1},\beta_{k-1},0\right)$ is a basis of $H(y,E_{3}^{\prime })$. Then, (10) yields $\beta_{i}=\varphi \left(\alpha_{i}\right)$. It is effortlessly demonstrated that $\alpha_{1},\ldots ,\alpha_{k-1}$ is a basis of $H(y_{1})$, and $\beta_{1},\ldots ,\beta_{k-1}$ is a basis of $H(y_{2})$. Thus,

$$\varphi \left(H\left(y_{1}\right)\right)=H\left(y_{2}\right).$$

Similarly, by $H(y,E_{4})\subseteq S$, we obtain

$$\psi \left(H\left(y_{1}\right)\right)=H\left(y_{2}\right).$$

Then, we have

$$\psi \left(H\left(y_{1}\right)\right)=H\left(y_{2}\right)=\varphi \left(H\left(y_{1}\right)\right)=H\left(y_{1}\right).$$

That is to say, $H(y_{1})$ is the ψ-invariantsubspace of $F_{q}^{k}$.

Let $\alpha_{1},\ldots ,\alpha_{k-1},\alpha_{k}$ be a basis of $F_{q}^{k}$, where $\alpha_{1},\ldots ,\alpha_{k-1}$ is a basis of $H(y_{1})$. Then, the matrix of ψ with respect to this basis is

$$B=\left(\begin{array}{cc} B_{1} & B_{2}\\ 0 & b \end{array}\right),$$

where $B_{1}$ is the matrix of $\psi |H(y_{1})$ with respect to $\alpha_{1},\ldots ,\alpha_{k-1}$. Note that *M* is the matrix of ψ with respect to the standard basis, and thus *M* and *B* are similar and have the same characteristic polynomial. So

$$f\left(x\right)=|xI-B_{1}|\left(x-b\right),$$

a contradiction with the irreducibility of f(x). Hence, $H(y,E_{3})⊈S$ or $H(y,E_{4})⊈S$. It is easy to see that $r(\left\{H\left(y,E_{1}\right)\cup H\left(y,E_{2}\right)\cup H\left(y,E_{3}\right)\right\}\setminus T_{0}^{\prime })=m-1$ or $r(\left\{H\left(y,E_{1}\right)\cup H\left(y,E_{2}\right)\cup H\left(y,E_{4}\right)\right\}\setminus T_{0}^{\prime })=m-1$. So, dimV(y,D)=m−1.

In conclusion, for any $y\in F_{q}^{m}\setminus \left\{0\right\}$, dimV(y,D)=m−1. By Lemma 4, C(D) is minimal. □

Combining Theorem 3 and Lemma 5, we have

**Corollary** **3.** *Let s≥4 and $\Phi =\{E_{1},\ldots ,E_{s}\}$ be a sunflower of $F_{q}^{m}$ with center $T_{0}^{\prime }$. If $\left\{E_{1},E_{2},E_{3},E_{4}\right\}$ are defined as (7), then C(D) is minimal.*

**Example** **2.** *Take q=5,k=2, and $t_{0}=1$. Let $f\left(x\right)=x^{2}+x+1$ and*

$$M=\left(\begin{array}{cc} 0 & -1\\ 1 & -1 \end{array}\right).$$

 *It is easily checked that $f\left(x\right)\in F_{q}\left[x\right]$ is an irreducible polynomial of degree 2 and the characteristic polynomial of M. Then, the code C(D) constructed based on Theorem 3 is a minimal ${\left[480,5\right]}_{5}$ linear code with minimum distance 300, and*

$$\frac{w_{\min}}{w_{\max}}=\frac{3}{4}<\frac{4}{5}.$$

Now, we construct some sunflowers Φ with center $T_{0}^{\prime }$ such that C(D) are not minimal. Let us recall from (6) that

$$E_{b}=\mathrm{Span}\left\{e_{1}+be_{k+1},e_{2}+be_{k+2},\ldots ,e_{k}+be_{2k},e_{2k+1},\ldots ,e_{m}\right\}.$$

Let

(13) $$\Phi =\{E_{b}|b\in F_{q}\}.$$

It is easy to see that Φ is a sunflower of $F_{q}^{m}$ with center $T_{0}^{\prime }$.

**Theorem** **4.** *For the sunflower Φ defined in (13), the linear code C(D) is not minimal.*

**Proof.**  Let $y_{0}=e_{1}$. Then, for any $b\in F_{q}$, we obtain

$$\begin{array}{cc} H(y_{0},E_{b}) & =\mathrm{Span}\{e_{2}+be_{k+2},\ldots ,e_{k}+be_{2k},e_{2k+1},\ldots ,e_{m}\}\\ & \subseteq \mathrm{Span}\{e_{2},\ldots ,e_{k},e_{k+2},\ldots ,e_{2k},e_{2k+1},\ldots ,e_{m}\}. \end{array}$$

By (3), we have

$$H\left(y_{0},D\right)\subseteq \mathrm{Span}\left(\left\{e_{2},\ldots ,e_{k},e_{k+2},\ldots ,e_{2k},e_{2k+1},\ldots ,e_{m}\right\}\right).$$

Then, $\dim V(y_{0},D)\leq m-2$. By Lemma 4, we have that c$\left(y_{0}\right)$ is not minimal and C(D) is not minimal. □

Combining Theorem 4 and Corollary 1, we have

**Corollary** **4.** *Let 3<s≤q and $S\subseteq F_{q}$ where #S=s. Let $\Phi =\{E_{b}|\;b\in S\}$. Then, C(D) is not minimal.*

**Remark** **2.** *In Theorem 3, Corollary 3, Theorem 4, and Corollary 4, the center of the sunflower Φ is the special subspace $T_{0}^{\prime }$. When the center is a general subspace, we have not yet proved the minimality of C(D).*

**Example** **3.** *Take q=3,k=2, and $t_{0}=2$. Then, the code C(D) constructed based on Theorem 4 is ${\left[216,6\right]}_{3}$ linear code with minimum distance 108, and*

$$\frac{w_{\min}}{w_{\max}}=\frac{2}{3}.$$

*According to Magma experiments, there exists $y_{1}=\left[1,0,0,0,0,0\right]\in F_{3}^{6}$ such that $\dim V(y_{1},D)=4$. Then, it follows from Lemma 4 that C(D) is not minimal.*

## 4. Concluding Remarks

In this paper, we use the approach used in [14] to study the minimality of linear codes constructed from sunflowers in all cases. In [23], the authors proved that if the number *s* of the elements in a sunflower satisfying s≥p+1, then the corresponding linear code over $F_{p}$ is minimal, where *p* is a prime number. Our results in this paper generalize [23] (Theorem 10). We discuss the minimality of linear codes constructed from sunflowers for all *s*. We obtain the following three results: (1) when s≥q+1, for any sunflower, the corresponding linear code is minimal; (2) when 2≤s≤3≤q, for any sunflower, the corresponding linear code is not minimal; (3) when 3<s≤q, for some sunflowers, the corresponding linear codes are minimal, whereas for some other sunflowers, the corresponding linear codes are not minimal.

## Data Availability

No new data were created or analyzed in this study. Data sharing is not applicable to this article.

## References

[1] Carlet C., Ding C., Yuan J. (2005). Linear codes from highly nonlinear functions and their secret sharing schemes. IEEE Trans. Inf. Theory.

[2] Chabanne H., Cohen G., Patey A., Lee H.-S., Han D.-G. (2014). Towards secure two-party computation from the wire-tap channel. Proceedings of the ICISC 2013.

[3] Ding C., Yuan J. (2003). Covering and secret sharing with linear codes. Discrete Mathematics and Theoretical Computer Science.

[4] Massey J.L. Minimal codewords and secret sharing. Proceedings of the 6th Joint Swedish-Russian Workshop on Information Theory.

[5] Yuan J., Ding C. (2006). Secret sharing schemes from three classes of linear codes. IEEE Trans. Inf. Theory.

[6] Ashikhmin A., Barg A., Cohen G., Huguet L., Cohen G., Giusti M., Mora T. (1995). Variations on minimal codewords in linear codes. Applied Algebra, Algebraic Algorithms and Error-Correcting Codes, (AAECC-11).

[7] Cohen G.D., Mesnager S., Patey A., Stam M. (2003). On minimal and quasi-minimal linear codes. Proceedings of IMACC.

[8] Ashikhmin A., Barg A. (1998). Minimal vectors in linear codes. IEEE Trans. Inf. Theory.

[9] Ding C., Fan C., Zhou Z. (2017). The dimension and minimum distance of two classes of primitive BCH codes. Finite Fields Appl..

[10] Zhou Z., Ding C. (2013). Seven Classes of Three-Weight Cyclic Codes. IEEE Trans. Commun..

[11] Ding C., Heng Z., Zhou Z. (2018). Minimal binary linear codes. IEEE Trans. Inf. Theory.

[12] Heng Z., Ding C., Zhou Z. (2018). Minimal linear codes over finite fields. Finite Fields Appl..

[13] Alfarano G.N., Borello M., Neri A. (2022). A geometric characterization of minimal codes and their asymptotic performance. Adv. Math. Commun..

[14] Lu W., Wu X. (2021). The parameters of minimal linear codes. Finite Fields Appl..

[15] Tang C., Qiu Y., Liao Q., Zhou Z. (2021). Full Characterization of Minimal Linear Codes as Cutting Blocking Sets. IEEE Trans. Inf. Theory.

[16] Alfarano G.N., Borello M., Neri A., Ravagnani A. (2022). Three Combinatorial Perspectives on Minimal Codes. Siam J. Discret. Math..

[17] Bartoli D., Bonini M. (2019). Minimal linear codes in odd characteristic. IEEE Trans. Inf. Theory.

[18] Bartoli D., Bonini M., Gunes B. (2021). An inductive construction of minimal codes. Cryptogr. Commun..

[19] Bartoli D., Cossidente A., Marino G., Pavese F. (2022). On cutting blocking sets and their codes. Forum Math..

[20] Bonini M., Borello M. (2021). Minimal linear codes arising from blocking sets. J. Algebr. Comb..

[21] Héger T., Nagy Z.L. (2022). Short minimal codes and covering codes via strong blocking sets in projective spaces. IEEE Trans. Inf. Theory.

[22] Gorla E., Ravagnani A. (2016). Equidistant subspace codes. Linear Algebra Its Appl..

[23] Li X., Yue Q., Tang D. (2022). A family of linear codes from constant dimension subspace codes. Des. Codes Cryptogr..

[24] Lu W., Wu X., Cao X., Luo G., Qin X. (2023). Minimal linear codes constructed from partial spreads. arXiv.

